# Veterinary Education

**Published:** 1900-12

**Authors:** 


### CORRESPONDENCE.

### VETERINARY EDUCATION.

Editor Journal of Comparative Medicine, etc.

Sir: The first veterinary school in this country was established in Boston nearly a half-century ago by Dr. George Dadd, and the veterinarians of New England have taken a keen interest in the progress of veterinary education from that time to this. We claim for this section the honor of having established the first three-year veterinary school in America, and of having led in work pertaining to the suppression of the animal diseases, of which foot-and-mouth disease, pleuro-pneumonia, glanders, and tuberculosis are notable examples.

Unfortunately, our leading position has received what may be a serious setback by the action of the Corporation of Harvard College in voting that no new students shall be received by the veterinary school. This action has caused a great deal of serious thought by veterinarians interested in the progress of their profession, and I should like to record the views of one who has devoted some attention to the education of men who wish to become veterinarians, and has observed, if he has had little experience in this line. I shall try to be brief, and while what I write is probably influenced to a certain extent by the change that has occurred in our sole New England school, my opinions are based upon prolonged observation. I should add that I am not and never have been connected in any way with Harvard University, and while I am not in full sympathy with all of the details of the administration of the Harvard Veterinary School, I recognize the vast impetus that this school has given to veterinary education not only in New England, but in all parts of the United States.

The Harvard Veterinary School is closed for want of public support. It is a sad reflection upon the value of veterinary work and upon the standing of the veterinary profession that in the richest part of the country and with the backing of the most famous American university, it has not been possible to secure support enough to keep the school going on an adequate basis. It is, however, to the credit of the University that it has refused to continue to conduct a school that does not attain to the highest standard of veterinary education in this country. The original mistake that was made appears to have been based on the supposition that a veterinary school required no equipment in addition to that of a good medical school excepting a hospital, a farriery, and a dissecting-room. Moreover, it was thought that a large part of the teaching of veterinary students could be conducted by professors in the school of medicine. The Veterinary School had no professors who were employed to devote all of their time to its interests. It has always been necessary for the teachers to divide their time between their classes and their outside pot-boiling work of a public or private nature. So the Veterinary School has not been able to secure the best energies of even one man; whereas, it should have had the undivided interest of at least five or six.

We have but to recall the history of the early days of the veterinary schools to find complete support for these views.

Shortly after the founding of the first veterinary school in France, in 1762, similar institutions were erected in nearly all civilized countries. There appeared to be at that time a very general impression that the work of the veterinarian was but a subordinate kind of medical practice, so most of the teaching of the early days was given into the hands of physicians and surgeons, some of whom had received special instruction in the veterinary schools of France, and some of whom were taken directly from the proper work of the medical profession. The work of the veterinary schools during the first half-century of their existence was most discouraging, and it was not until there appeared a new generation of veterinarians who had been educated by veterinarians that their science took its proper place in the economy of nations. Of course, it is true that some of the greatest steps in the development of veterinary education were taken by men who were physicians; but these men were not practising physicians nor teachers in medical schools, they were physicians or surgeons who were also veterinarians, as Wolstein, who established the veterinary school in Vienna, and, indeed, was the founder of scientific veterinary work in Germany. While Wolstein was originally a physician, he later devoted all of his time to veterinary work, and deserves to rank as the most eminent veterinarian of his day.

At present the successful veterinary schools of the world are under the direction of veterinarians, and their students are taught by veterinarians who devote all their time to this work. A little consideration will show that success cannot be achieved in any other way. As Dr. Reynolds, of Minnesota, has said, "It would be as reasonable to put carpentry work under the supervision of blacksmiths and blacksmithing under the supervision of carpenters as to subject veterinary work to medical control."

Physicians who teach in medical colleges and are also asked to instruct veterinary students do not and cannot furnish the detailed special instruction and point out the practical applications that the veterinarian must have. Take the subject of physiology, for example. A physiologist teaching in a medical school will say that the principles of physiology are of universal application, and that his course of physiology is as good for a veterinarian as it is for a medical student. Probably, if the veterinary student were intending to become a specialist in physiology, a first-class course in a good medical school would serve for a foundation. But the practising veterinarian ought to be able, and if he is to succeed fully he must be able, to apply his physiology in a thousand ways that do not come before the physician, and that are entirely unknown to the medical professor of physiology. The veterinarian must know about the respiratory mechanism and needs of each species of domestic animals; he must know about the nutritive needs of animals kept for different purposes and doing different kinds of work; he must know how to utilize to the best advantage the common foodstuffs of the farm for growing calves for beef or milk, for producing eggs, and for sustaining the draught-horse and the trotter. The veterinarian must very thoroughly understand the physiology of the reproductive processes of all kinds of animals, the laws of heredity, and the influences and conditions that may be applied to improve the offspring. These are but a very few of the many points that would require a volume for their appropriate discussion. As much could be said in favor of separate special courses by and for veterinarians in pathology, bacteriology, pharmacology, physiological chemistry, histology, etc.

The need for separate instruction to veterinary students in each of these subjects may not at first be clear to one who is not a veterinarian; but when we recall that the subject of pathology, for example, has developed to such an extent that it is now divided into many specialties, and the appropriate consideration of any one of these specialties requires work for a lifetime, we can readily understand that the veterinary student cannot possibly obtain during his brief college course as much special pathology of animals as he ought to have, even if all of the time that he can devote to pathology is given to the study of the special parts that he absolutely must know; then we can realize the urgent need for special application and special instruction.

A great mistake has been made in denominating veterinary schools as schools of veterinary medicine. This is usually interpreted to mean that veterinary science is a branch of medicine. Nothing could be further from the truth. Upon this false conception should be placed a large share of the responsibility for the tardy development of veterinary education in this country. It is true that the veterinary sciences draw largely upon the medical sciences, or, at least, the sciences that are usually so termed. As a matter of fact—and this thought has been partly developed by Dr. Theobald Smith—veterinary schools are schools of applied biology, and are not more dependent upon schools of medicine than are other schools for biological instruction. If a veterinary school must be attached to some other institution, it is very much more logical, and history shows that it is much more successful, to combine veterinary science and agriculture than it is to combine veterinary science and medicine. The veterinarian is not a second-rate "doctor," nor does he belong to a subordinate branch of the medical profession. He is the natural and highest authority on questions pertaining to animal husbandry. Most of the great questions of animal husbandry and of veterinary science are not *medical* questions at all. As Dr. Huidekoper has said, "the chief use of the veterinarian in the army is not that of a tinkerer to treat colic and sew up wounds;" not at all. That veterinarian can be most valuable to the army and to the State and to the nation who is best prepared to take a comprehensive view of his science, and so advise in regard to the breeding, the feeding, and the care of animals that they will be made stronger to endure work and resist disease, and in this way lengthen the average life, or productivity, or lessen the cost of rearing and maintenance a great deal more than these could be improved by the work of the veterinarian who gets his training in a branch of a medical school and who must regard tinkering as the chief end of his profession. Dr. Pearson has tried, although he has not succeeded fully, to express this thought by the use of the title "animal engineer."

The day of the veterinary branch of a medical school, or of the veterinary school devoted almost exclusively to medical teaching, was past before the Harvard School of Veterinary Medicine was opened. It is a pity that Harvard University could not have seen this, and thus avoided its present embarrassment; but this experience has at least one great advantage. It shows that the veterinary school comprises something that is entirely aside, apart from, and beyond veterinary *medicine*. It shows that a school of veterinary *medicine*

will not succeed, even when attached to the best medical school in the country and backed by the greatest University of the land. It is to be hoped that Harvard University will now establish a school of veterinary *science* wherein instruction will be afforded in everything that pertains to the domestic animals. Such a school should have as part of its equipment a farm to be used as a breeding-station. This part of the equipment of a veterinary school is now recognized as indispensable, and the most enlightened members of the veterinary profession are on record as recognizing the indispensable need for this equipment in a resolution adopted unanimously at the last International Veterinary Congress (Baden-Baden, 1899).

The subject of physiology should be studied practically upon all domestic animals. This does not mean vivisection. The instruction in physiology and in breeds and breeding and in animal husbandry and zoötechnics should be at least as complete as it is in the best agricultural college in the country.

This new institution should have sufficient resources to justify the employment of the entire time of five or six members of the teaching staff. An endowment of at least $500,000 is necessary to the maintenance of a creditable veterinary school. Here is an opportunity for a philanthropist. YANKEE.

---

ON the morning of November 13th the central or main laboratories of bacteriology and histology of the New York State Veterinary College were burned out, with all their valuable contents.

Fortunately, the research laboratories of the same department, and situated on the same floor, were saved. The whole of the basement with the first and second floors, including museum, offices, lecture-room, and laboratories of physiology, materia medica, and pharmacy escaped. The east wing, ninety feet long, with amphitheatre, dissecting-room, preparing-room, locker-room, laboratories, and small animal-house, remained unscathed, as did also the separate buildings, the mortuary, bone-room, operating-room, house of stud groom, and three clinical buildings.

Work was begun at once on the burnt portion, and by the 17th it had been cleared and roofed in, and from the 19th work will go on fully in all departments as before. The first floor of the museum has been fitted up for a laboratory of histology and embryology, and a periodical-room, the spaces between the museum cases being availed of; the former periodical-room and part of the second floor of the museum are devoted to bacteriology and pathology, while in both departments the research laboratories are still available.

The short course for practitioners advertised to commence January 3d will be carried out as formerly provided for, with ample facilities.

